# The epidemiologic characteristics of healthcare provider-diagnosed eczema, asthma, allergic rhinitis, and food allergy in children: a retrospective cohort study

**DOI:** 10.1186/s12887-016-0673-z

**Published:** 2016-08-20

**Authors:** David A. Hill, Robert W. Grundmeier, Gita Ram, Jonathan M. Spergel

**Affiliations:** 1 Department of Pediatrics, The Children’s Hospital of Philadelphia, Philadelphia, PA USA; 2 Division of Allergy and Immunology, The Children’s Hospital of Philadelphia, 3550 Market St., Philadelphia, PA 19104 USA; 3 Department of Biomedical and Health Informatics, The Children’s Hospital of Philadelphia, Philadelphia, PA USA; 4 Institute for Immunology, Perelman School of Medicine at the University of Pennsylvania, Philadelphia, PA USA

**Keywords:** Eczema, Allergic rhinitis, Asthma, Food allergy, Prevalence

## Abstract

**Background:**

The rates of childhood allergic conditions are changing, prompting the need for continued surveillance. Examination of healthcare provider-based diagnosis data is an important and lacking methodology needed to complement existing studies that rely on participant reporting.

**Methods:**

Utilizing our care network of 1,050,061 urban and sub-urban children, we defined two retrospective cohorts: (1) a closed birth cohort of 29,662 children and (2) a cross-sectional cohort of 333,200 children. These cohorts were utilized to determine the epidemiologic characteristics of the conditions studied. Logistic regression was utilized to determine the extent to which food allergy was associated with respiratory allergy.

**Results:**

In our birth cohort, the peak age at diagnosis of eczema, asthma, rhinitis, and food allergy was between 0 and 5 months (7.3 %), 12 and 17 months (8.7 %), 24 and 29 months (2.5 %), and 12 and 17 months (1.9 %), respectively. In our cross-sectional cohort, eczema and rhinitis prevalence rates were 6.7 % and 19.9 %, respectively. Asthma prevalence was 21.8 %, a rate higher than previously reported. Food allergy prevalence was 6.7 %, with the most common allergenic foods being peanut (2.6 %), milk (2.2 %), egg (1.8 %), shellfish (1.5 %), and soy (0.7 %). Food allergy was associated with development of asthma (OR 2.16, 95 % CI 1.94-2.40), and rhinitis (OR 2.72, 95 % CI 2.45-3.03).

**Conclusions:**

Compared with previous reports, we measure lower rates of eczema and higher rates of asthma. The distribution of the major allergenic foods diverged from prior figures, and food allergy was associated with the development of respiratory allergy. The utilization of provider-based diagnosis data contributes an important and lacking methodology that complements existing studies.

**Electronic supplementary material:**

The online version of this article (doi:10.1186/s12887-016-0673-z) contains supplementary material, which is available to authorized users.

## Background

Eczema, asthma, and allergic rhinitis (rhinitis) are among the most common childhood medical conditions in the United States [1]. The large disease burden of these conditions represents a significant source of morbidity, mortality, and healthcare expenditures [2, 3]. Additionally, disease rates for these conditions are thought to be changing [4–6], prompting the need for continued surveillance. While prior studies aimed at determining the epidemiologic characteristics of these conditions have provided important insights, the majority of large-scale studies have relied on participant reporting as opposed to provider-based diagnosis methodology [1, 6–9]. While studies that utilize participant reporting are a valuable tool when studying changing disease patterns across large populations, they have limitations including participant honesty, introspective ability, and understanding of complex disease processes. Indeed, it has been shown that studies that utilize participant reporting or provider-based diagnosis data provide different estimates of disease patterns [10, 11]. As such, provider-based diagnosis data is needed to complement existing studies and to provide for the most accurate estimates of disease rates.

Like eczema, asthma, and rhinitis, allergies to foods are common in the United States and their prevalence may be changing [4, 12]. Childhood food allergy is associated with impaired quality of life, limited social interactions, comorbid allergic conditions, and significant economic cost [13–18]. Importantly, a severe allergic reaction resulting in anaphylaxis can be life threatening, and food allergens are the most common cause of anaphylaxis and anaphylaxis-related mortality in children and adolescents [19]. Recent estimates have reported food allergy prevalence figures between 4 and 8 %, however, these studies are limited in size and scope or rely on participant reporting rather than healthcare provider-based diagnosis [18, 20, 21]. Interestingly, prior studies have suggested an association between food allergy and the development of comorbid allergic conditions, though these studies have been relatively small in size, examined only a single comorbid condition, or relied on participant reporting methodology [16, 18]. An examination of the epidemiologic landscape of food allergy, through the utilization of healthcare provider-based diagnosis data, is therefore needed.

We describe a retrospective analysis of a primary care network of 1,050,061 children. We define two large primary care cohorts: (1) a closed birth cohort of 29,662 children followed continuously in our primary care practices from age < 30 days through their 5th birthday and (2) a larger cross-sectional cohort of 333,200 children who established primary care in our network before their 18th birthday and had at least 12 months of follow-up in our network. We examine healthcare provider-based diagnosis data to determine the age at diagnosis, incidence, and prevalence of eczema, asthma, rhinitis, and food allergy. We further examine asthma-related medication prescriptions to more accurately measure asthma prevalence rates. Finally, we exploit the large number of patients in our cohorts to determine the extent to which food allergy is associated with the development of asthma, and rhinitis. This study complements existing studies, identifies different disease rates than previously reported, and provides important information that will shape future efforts aimed at prevention, diagnosis, and management of these common pediatric conditions.

## Methods

### Cohort design

The Children’s Hospital of Philadelphia care network is both an international referral center and a provider of primary and sub-specialty care services to patients residing in Pennsylvania, New Jersey, and Delaware. Our primary care network, which encompasses more than 30 sites, has been validated as an accurate tool for estimating disease rates across our broader community population [22]. We extracted data from The Children’s Hospital of Philadelphia’s electronic medical records (EMRs) of 1,050,061 children who obtained any primary, specialty, or hospital-based care in our health system. An honest broker removed direct patient identifiers (e.g., medical record number, name, etc.) to create a dataset with limited identifiers (e.g., date of birth, dates of healthcare encounters, diagnoses, etc.). Data analyses tasks were completed using R version 3.1.2. (Wien, Austria). The data extraction included data on healthcare provider-diagnosed conditions for 379,134 children who received outpatient healthcare before their 18th birthday at a primary care location between 1/1/2001 and 12/31/2013. To determine age at diagnosis and incidence rates in the first 5 years of life, we generated a closed birth cohort by identifying 29,662 children who established healthcare in our primary care network before age 30 days and were followed in our network at least through 5 years of age (birth cohort). To determine prevalence rates, we generated a cross-sectional cohort by excluding 45,934 children who received care for less than 12 months, leaving 333,200 children who received one or more years of care in our primary care network (cross-sectional cohort). Individual children with each diagnosis were enumerated. The Institutional Review Board of The Children’s Hospital of Philadelphia reviewed the retrospective cohort portion of our study and determined that it did not meet the definition of “human subject” research and was therefore exempt from requiring ethics approval.

### Definitions of conditions studied

Each cohort was examined for the presence of International Classification of Diseases Ninth Revision (ICD-9) diagnosis codes that fell into one of three diagnosis groups: eczema, asthma, or allergic rhinitis (rhinitis) (Additional file 1: Table S1). We identified children with specific food allergies using the allergy module of our EMR (Epic Systems, Verona, WI) (Additional file 2: Table S2). For our principal analysis of eczema, asthma, and rhinitis, we required patients to have diagnosis codes representative of each condition during at least two separate care visits occurring at least six months apart. As a sensitivity analysis to assess the potential impact of alternative definitions, we utilized a subset of our cross-sectional cohort with at least 24 months of follow-up (*n* = 296,556) to identify those children whose eczema, asthma, and rhinitis diagnoses were present in at least two separate care visits occurring two or more years apart. To minimize the likelihood of including non-allergic wheeze in our analysis, we excluded diagnosis codes relating to reactive airway and post-viral wheeze (Additional file 3: Table S3), as well as any asthma diagnoses made before the age of 1 year of life. Finally, to minimize the likelihood of including lactose intolerance and gluten sensitivity (celiac disease) in our analysis, we re-coded patients with diagnosis codes corresponding to these conditions as non-milk or non-wheat allergic, respectively.

### Query validation via chart review

To determine the extent to which ICD-9 diagnosis codes and food allergy data correctly represented the presence of each of the conditions of interest, charts of 240 subjects were manually reviewed and estimations of the accuracy of the coded EMR data were made. To establish accuracy, diagnoses were compared to practice parameters and consensus guidelines from the Joint Task Force on Practice Parameters (representing the AAAAI, ACAAI, and the JCAAI) [23–26]. The chart review portion of our study was approved by The Institutional Review Board of The Children’s Hospital of Philadelphia and performed under IRB protocol number 07-005420_AM13.

### Analysis of asthma medication prescriptions

Data on asthma-related medication prescriptions was extracted from the medical records of patients who fell into the asthma diagnosis group. After excluding asthma diagnoses and medications in the first year of life, we compared the rates of asthma based on definitions that required either (a) the occurrence of at least two visits with an asthma-related diagnosis code (ICD-9 codes 493.00 to 493.92) occurring at least six months apart; (b) prescriptions for asthma medications on at least two separate dates; or (c) both a diagnosis code and prescriptions.

### Analysis of food allergy as a risk factor for development of respiratory allergy

Utilizing our birth cohort, we performed a risk analysis to determine the extent to which food allergy in general, and allergy to specific foods, predisposed to the development of respiratory allergy (asthma or rhinitis). For the outcome of asthma, we utilized our most restrictive definition (two asthma diagnoses at least six months apart, and asthma-related medication prescriptions on at least two separate dates). Asthma and rhinitis were analyzed as independent outcomes. For each outcome, we excluded children who developed the outcome prior to the onset of food allergy. Outcomes were assessed using logistic regression with adjustment for gender, race, and ethnicity. Adjusted odds ratios and 95 % confidence intervals (CI) were reported.

### Availability of data and materials

The dataset supporting the conclusions of this article is available in the Zenodo repository, http://dx.doi.org/10.5281/zenodo.44529.

## Results

### Cohort demographics

The demographic characteristics of patients in both cohorts are shown in Table 1. Our patient population were found to be approximately 48 % white, 40 % black, and have a primarily non-Medicaid payer type. To determine the accuracy of our coded EMR data, a manual chart review was performed on a subset of charts. The percent confirmed diagnosis for all conditions studied was 92 % (Additional file 4: Table S4).

**Table 1:** Demographic characteristics of birth and cross-sectional cohorts

	Frequency, % (n)	
Characteristic	Birth	Cross-sectional
	(29,662)	(333,200)
*Race/ethnicity*		
White	48 (14,188)	55 (183,308)
Black	40 (11,967)	29 (97,795)
Asian or Pacific Islander	3 (825)	3 (9,152)
Other	1 (166)	1 (2,005)
Unknown	8 (2,516)	12 (40,940)
Non-Hispanic	97 (28,661)	95 (317,868)
Hispanic	3 (1,001)	5 (15,332)
*Gender*		
Male	51 (15,044)	51 (169,032)
Female	49 (14,618)	49 (164,168)
*Payer type*		
Medicaid	33 (9,885)	26 (86,860)
Non-medicaid	67 (19,777)	74 (246,340)
*Birth year*		
Before 2000	0 (0)	39 (128,864)
2000 to 2004	20 (5,861)	25 (83,133)
2005 to 2009	80 (23,801)	25 (83,519)
2010 to 2013	0 (0)	11 (37,684)

### Age at diagnosis and overall incidence

The incidence of eczema, asthma, and rhinitis in each 6-month age strata of our birth cohort are shown in Table 2. The incidence of eczema during the first 5 years of life was 15.3 %, with a peak age at diagnosis between 0 and 5 months of life (7.3 %). The incidence of asthma during the first 5 years of life was 22.4 %, with a peak age at diagnosis between 12 and 17 months (8.7 %). The incidence of rhinitis over the first 5 years of life was 17.2 %, with a peak age at diagnosis between 24 and 29 months (2.5 %).

**Table 2:** Age at diagnosis of eczema, asthma, and rhinitis

	Frequency, % (n)		
Age range, months	Eczema	Asthma	Rhinitis
0 - 5	7.3 (2,169)	0.0 (0)	0.1 (25)
6 - 11	3.1 (929)	0.0 (0)	0.9 (281)
12 - 17	1.8 (524)	8.7 (2,583)	1.7 (497)
18 - 23	1.0 (289)	3.0 (887)	2.0 (607)
24 - 29	0.7 (204)	2.5 (756)	2.5 (730)
30 - 35	0.4 (109)	1.9 (563)	2.0 (606)
36 - 41	0.5 (134)	2.1 (614)	2.4 (721)
42 - 47	0.2 (68)	1.6 (484)	1.8 (542)
48 - 53	0.2 (66)	1.6 (477)	2.2 (659)
54 - 59	0.1 (37)	1.0 (295)	1.4 (420)
Total	15.3 (4,529)	22.4 (6,659)	17.2 (5,088)

**Fig. 1:**
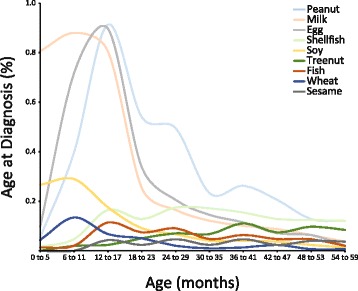
Age at diagnosis of common food allergies

The incidence of food allergy in each 6-month age strata is shown in Fig. 1. The incidence of at least one food allergy during the first 5 years of life was 8.2 %, with a peak age at diagnosis between 12 and 17 months of life (1.9 %). The incidence of peanut, milk, egg, and soy allergies over the first 5 years of life ranged from 1.1 to 3.4 %, with a peak age at diagnosis between 12 and 17 months for peanut or egg allergy and between 6 and 11 months for milk or soy allergy. The incidence of shellfish allergy was 1.2 %, with a peak age at diagnosis between 24 and 29 months.

### Prevalence

Prevalence of eczema, asthma, and rhinitis by age strata in our cross-sectional cohort is shown in Table 3. The prevalence of eczema across all ages in our cohort was 6.7 %, with the peak prevalence between 3 and 5 years of life (7.7 %). The prevalence of asthma across all ages in our cohort was 21.8 %, with the peak prevalence between 14 and 17 years of age (23.0 %). The prevalence of rhinitis across all ages in our cohort was 19.9 %, with the peak prevalence between 14 and 17 years (24.8 %). As a sensitivity analysis to assess the potential impact of alternative disease definitions, we utilized a subset of our cross-sectional cohort with at least 24 months of follow-up (*n* = 296,556) to identify those children whose eczema, asthma, and rhinitis diagnoses were present in at least two separate care visits occurring two or more years apart. The prevalence of eczema, asthma, and rhinitis did not substantially change when we required diagnosis codes to be present for at least two separate care visits occurring at least two years apart (7.1, 20.7, and 18.2 %, respectively).

**Table 3:** Prevalence of eczema, asthma, and rhinitis

	Frequency, % (n)		
Age range, years (n)	Eczema	Asthma	Rhinitis
0 - 3 (187,039)	7.3 (13,564)	12.3 (23,078)	5.6 (10,466)
3 - 5 (195,200)	7.7 (14,936)	19.4 (37,903)	13.7 (26,709)
6 - 10 (199,287)	6.9 (13,826)	22.9 (45,666)	21.7 (43,255)
11 - 13 (143,392)	4.8 (6,845)	22.6 (32,371)	23.3 (33,478)
14 - 17 (107,717)	3.9 (4,228)	23.0 (24,822)	24.8 (26,701)
All ages (333,200)	6.7 (22,464)	21.8 (72,534)	19.9 (66,390)

**Fig. 2:**
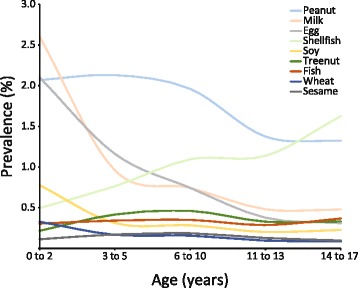
Prevalence of common food allergies

Prevalence of food allergies by category and age strata in our cross-sectional cohort is shown in Fig. 2. The prevalence of at least one food allergy across all ages in our cohort was 6.7 %, with the peak prevalence between 0 and 3 years of life (5.7 %). Peanut allergy was most common (2.6 %), followed by milk (2.2 %), egg (1.8 %), shellfish (1.5 %), and soy (0.7 %). Milk, egg, and soy allergy were most common between 0 and 3 years of age (2.6, 2.1, and 0.8 % respectively), while peanut allergy was most common between 3 and 5 years (2.1 %) and shellfish allergy was most common between 14 and 17 years (1.6 %).

### Examination of asthma-related medication prescriptions

Given that asthma rates were elevated in our population compared with previous reports, we sought to more accurately determine asthma prevalence in our population by correlating ICD-9 codes with asthma-related medication prescriptions. Our most stringent asthma condition inclusion criteria required the occurrence of at least two encounters after the first birthday with an asthma diagnosis code separated by at least six months, and the presence of prescriptions for one or more asthma medications on at least two separate dates. Based on these criteria, we estimated an asthma prevalence rate of 21.6 % in our birth cohort and 18.4 % in our cross-sectional cohort (Additional file 5: Table S5). In a manual review of charts of 20 children meeting our ICD-9 code definition of asthma (two visits at least 6 months apart), 18 (90 %) were confirmed to have asthma. Of the 18 children with confirmed asthma, 2 (11 %) outgrew their asthma symptoms during the time they received treatment from our health system.

### Food allergy as a risk factor for respiratory allergy

Prior studies have suggested that patients with food allergy are at increased risk of developing asthma, however, these studies relied on participant reporting, were relatively small, or were limited to a single outcome [16, 18]. Utilizing our birth cohort, we sought to investigate the extent to which healthcare provider-diagnosed food allergy placed children at risk for developing respiratory allergy (asthma or rhinitis). The prevalence of asthma in patients with food allergy is shown in Table 4. Of patients with an established diagnosis of food allergy, 35 % went on to develop asthma (adjusted odds ratio: 2.16, 95 % CI: 1.94-2.4). Of the major food allergens, allergy to peanut, milk, and egg significantly predisposed to developing asthma (OR 1.74, 1.38, and 1.60, respectively). Additionally, patients with multiple food allergies were at increased risk of developing asthma as compared to patients with a single food allergy (Table 4).

**Table 4:** Asthma risk in patients with food allergy

Exposure	Asthma (%)	OR	95 % CI	p
Food allergy	35	2.16	1.94 - 2.40	5.8E-45
Peanut	31	1.74	1.31 - 2.28	8.8E-05
Milk	26	1.38	1.09 - 1.73	5.7E-03
Egg	29	1.60	1.12 - 2.27	8.8E-03
1 food	26	1.43	1.23 - 1.64	1.5E-06
2 foods	40	2.75	2.20 - 3.42	3.0E-19
3 foods	49	3.94	2.89 - 5.37	3.9E-18
4 foods	56	5.44	3.40 - 8.78	2.2E-12

The prevalence of rhinitis in patients with food allergy is shown in Table 5. Of patients with an established diagnosis of food allergy, 35 % went on to develop rhinitis (adjusted odds ratio: 2.72, 95 % CI: 2.45-3.03). Of the major food allergens, allergy to peanut, milk, and egg significantly predisposed to developing rhinitis (OR 2.59, 1.46, and 1.80, respectively). Additionally, patients with multiple food allergies were at increased risk of developing rhinitis as compared to patients with a single food allergy (Table 5).

**Table 5:** Rhinitis risk in patients with food allergy

Exposure	Rhinitis (%)	OR	95 % CI	p
Food allergy	35	2.72	2.45 - 3.03	2.0E-76
Peanut	35	2.59	1.99 - 3.34	5.1E-13
Milk	24	1.46	1.15 - 1.85	1.9E-03
Egg	28	1.80	1.25 - 2.55	1.3E-03
1 food	26	1.76	1.52 - 2.03	1.5E-14
2 foods	42	3.64	2.94 - 4.50	1.2E-32
3 foods	50	4.93	3.64 - 6.69	8.0E-25
4 foods	59	7.05	4.48 - 11.23	6.1E-17

## Discussion

We report the largest study to date to examine the epidemiologic characteristics of healthcare provider-diagnosed eczema, asthma, allergic rhinitis, and food allergy in a pediatric primary care population. Part of our analysis utilized a closed birth cohort of 29,662 children to determine the age at diagnosis and overall incidence rates for the conditions studied. This cohort was also used to determine the extent to which food allergies influence a child’s risk for developing respiratory allergy. We believe that, with the adoption of EMRs by most major academic medical centers, the utilization of healthcare provider-based diagnosis data within virtual birth cohorts will become a more common and powerful epidemiologic tool for the study of pediatric disease patterns and associations.

We found that the prevalence of rhinitis in our population was 19.9 %, a rate which is similar to previously reported figures [1, 7, 10]. In contrast, we found the prevalence of eczema in our population to be 6.7 %, a rate lower than previous reports [1, 8]. To minimize the likelihood of measurement error in our study we utilized healthcare provider-based diagnoses, which complement published data that utilize participant reporting methodologies, to ensure the most accurate estimation of disease burdens [10, 11]. We also restricted our analysis to diagnosis codes that were present on at least two separate visits that were at least six months apart to reduce the likelihood of including accidental or inaccurate diagnosis, and performed a chart review to confirm accuracy for ICD9 codes included in our study. It is possible that our eczema definition was less inclusive than previously published studies, or that infants outgrew their eczema within six months of diagnosis and were not counted. While consistent with global data that suggest eczema rates may be leveling off or decreasing in some regions [27], further investigation is necessary to determine whether eczema rates are changing in our population.

The prevalence of asthma in our cohort was measured to be 21.8 %, a rate higher than those reported previously, irrespective of study methodology (participant reporting or provider-based diagnoses) [1, 3, 4, 6]. To further define children with asthma, we correlated asthma-specific ICD-9 codes with asthma medication prescriptions in both our birth and cross-sectional cohorts. Using this approach, we estimated the prevalence rate of asthma in our population to be between 18.4 and 21.6 %. We utilized multiple methods to ensure that our asthma measurements were not over-estimates such as excluding all reactive airway disease diagnosis codes, excluding asthma diagnosis codes present before 1 year of life, performing a sub-analysis in which we required two asthma-specific diagnoses codes at least 2 years apart, and correlating asthma diagnosis codes with asthma-related medication prescription data. In a longitudinal chart review of children with asthma identified via ICD9 codes, we found that a large proportion (89 %) of children continued to display asthma symptoms, and/or require asthma-related medications, suggesting that providers were not “miss-coding” asthma that had resolved. Based on our findings, it may be that asthma rates in some populations of the country are higher than previously appreciated. Consistent with this, the asthma prevalence rates we measured are similar to those observed in other industrialized nations such as Australia, Singapore, and the United Kingdom [5, 9].

Current prevalence rates of food allergy are thought to range from 4 to 8 % [18, 20, 21]. We observed the overall prevalence of allergy to any food to be 6.7 %, a rate similar to those previously published [21]. However, the rates of food allergies to peanut, milk, egg, shellfish, and soy were proportionally higher in our patient population compared with prior reports [21]. Additionally, allergy to wheat is rarer than previously appreciated, while allergy to sesame is likely more common than previously appreciated and should be considered one of the common allergenic foods. We excluded lactose intolerance and gluten sensitivity from our analysis, a difference in methodology that may allow for more specific disease identification as compared previously published studies. Additionally, regional differences in the most common food allergens could be due to genetic or environmental factors. Additional studies are necessary to determine whether the rates of food allergy observed in our study are similar to those of other U.S. patient populations, or represent shifting epidemiologic trends.

Prior studies have suggested that patients with food allergy are at increased risk of developing comorbid allergic conditions such as asthma [16, 18]. We found that patients with existing food allergy were at increased likelihood of developing asthma, and rhinitis. The asthma and rhinitis rates observed in our food allergic population are approximately double those we observed in the general population, and our data represent stronger comorbid associations than those previously described [16, 18]. We also found that allergy to peanut, milk, and egg significantly increased risk of developing respiratory allergy, and that children with multiple food allergies were at increased risk of developing respiratory allergy when compared with children with a single food allergy. The strong association between food allergy and the subsequent development of asthma and/or rhinitis in our study may be due to the large size of our study cohort, or the utilization of healthcare provider-based diagnosis codes. Based on these findings, providers may choose to modify their counselling and management of patients with food allergy. Finally, our cohorts include data on other predisposing factors, outcomes, as well as data on healthcare utilization which can be examined in future studies.

### Limitations

This study was a secondary analysis of health records at a single institution collected as part of routine care. We relied primarily on diagnosis codes to identify conditions of interest, and choice of diagnosis codes result in potential biases in our data collection, and may be affected by billing or administrative constraints. However, in a chart review of a subset of our cohort in which we compared diagnosis codes to commonly accepted practice parameters and consensus guidelines, we found a high degree of diagnosis code accuracy. Furthermore, in our analysis of asthma-related medication prescription data we found that asthma-related diagnosis codes highly correlated with prescription practices. To ensure a suitable number of subjects for analysis, and capture the most common age at diagnosis for the conditions studied, the maximum age of inclusion in our birth cohort was set at 5 years. As such, a peak incidence occurring beyond 5 years could not have been detected by this study. Diagnoses corresponding to both IgE and non-IgE-mediated food allergies were included in our analyses and therefore, our food allergy data should not be interpreted as representing purely IgE-mediated food allergy. Additionally, it is not routine for patients to undergo food challenge or skin-prick testing to establish diagnosis of food allergy or allergic rhinitis, respectively. As such, an over-estimation of the prevalence of these conditions could occur. We examine disease prevalence rates in our healthcare system between 1/1/2001 and 12/31/2013 with the aim of establishing accurate prevalence data for the conditions studied. Longitudinal analysis of prevalence rates was not performed and as such we cannot draw conclusions on changing prevalence rates over time, and variations in prevalence between our study and other studies could be influenced by methods of data collection. Finally, our study cohort consists primarily of children residing in urban and suburban settings. Consequently, results in other communities, especially those that are rural, may differ.

## Conclusions

We utilized one of the largest pediatric primary care cohorts ever assembled to describe the epidemiologic characteristics of healthcare provider-diagnosed eczema, asthma, allergic rhinitis, and food allergy. Our study provides an important addition to existing studies that utilize participant reporting methodologies. In comparison with published rates, we measure lower rates of eczema, and higher rates of asthma, in our population. Additionally, rates of food allergies to peanut, milk, egg, shellfish, and soy may be proportionally higher than previously reported. Finally, our findings indicate that the presence of food allergy is a risk factor for the subsequent development of respiratory allergy. These findings allow new insights into the epidemiologic characteristics of these diseases, describe the importance of utilizing provider-diagnosis data to complement participant reporting methodologies, and provide important information to shape future efforts aimed at prevention, diagnosis, and management of these common pediatric conditions.

## Additional files

Additional file 1: Table S1. ICD9 codes by diagnosis group; A table of ICD9 codes used to define each diagnosis group. (PDF 318 kb) Additional file 2: Table S2. Food allergy codes by allergen; A table of food allergy codes used to define allergy to each food allergen. (PDF 258 kb) Additional file 3: Table S3. ICD9 codes excluded from our analysis; A table of ICD9 codes that were excluded from our analysis. (PDF 210 kb) Additional file 4: Table S4. Percent confirmed diagnosis on chart review; A table indicating the percent confirmed diagnosis on manual chart review. (PDF 199 kb) Additional file 5: Table S5. Analysis of asthma medication prescriptions; A table summarizing our analysis of asthma medication prescription practices and diagnosis codes. (PDF 208 kb)

## Abbreviations

CI: Confidence Interval
EMR: Electronic Medical Record
ICD: International Classification of Diseases
