# Supplementary material for: The epidemiologic characteristics of healthcare provider-diagnosed eczema, asthma, allergic rhinitis, and food allergy in children: a retrospective cohort study
Source: BMC Pediatr. 2016 Aug 20;16:133. doi: 10.1186/s12887-016-0673-z (PMC4992234; doi:10.1186/s12887-016-0673-z)
Supplement: Additional file 1: Table S1. — ICD9 codes by diagnosis group; A table of ICD9 codes used to define each diagnosis group. (PDF 318 kb) [file 12887_2016_673_MOESM1_ESM.pdf]

Table S1 ICD9 codes by diagnosis group

Diagnosis Group	ICD9 Code	Diagnosis Name	n
Eczema	691	Atopic dermatitis and related conditions	682
Eczema	691.8	Other atopic dermatitis and related conditions	18034
Eczema	691.8	Atopic dermatitis	11132
Eczema	691.8	Atopic eczema	468
Eczema	691.8	Atopic dermatitis and related conditions	195
Eczema	691.8	AD (atopic dermatitis)	38
Eczema	691.8	Mild atopic dermatitis	29
Eczema	691.8	Atopic dermatitis and related condition	10
Eczema	691.8	Dermatitis, atopic	3
Eczema	691.8	Papular atopic dermatitis	3
Eczema	691.8	Atopic dermatitis, mild	1
Eczema	691.8	Flexural eczema	1
Asthma	493.00	Extrinsic asthma, unspecified	23377
Asthma	493	Asthma	472
Asthma	493.00	Extrinsic asthma	385
Asthma	493.0	Extrinsic asthma	139
Asthma	493.00	Asthma, extrinsic	31
Asthma	493.00	Asthma with allergic rhinitis	11
Asthma	493.00	Asthma in pediatric patient	8
Asthma	493.00	Asthma, extrinsic, without status asthmaticus	7
Asthma	493.00	Childhood asthma	4
Asthma	493.00	Mild intermittent asthma without complication in pediatric patient	3
Asthma	493.00	Pollen asthma	2
Asthma	493.00	Asthma with hay fever	1
Asthma	493.00	Hay fever with asthma	1
Asthma	493.00	Mild persistent allergic atopic asthma without complication	1
Asthma	493.00	Mild persistent asthma without complication in pediatric patient	1
Asthma	493.00	Moderate persistent asthma with allergic rhinitis without complication	1
Asthma	493.00	Moderate persistent asthma with allergic rhinitis without status asthmaticus without complication	1
Asthma	493.00	Moderate persistent asthma without complication in pediatric patient	1
Asthma	493.00	Samter's triad	1
Asthma	493.01	Extrinsic asthma with status asthmaticus	799
Asthma	493.01	Asthma, extrinsic with status asthmaticus	6
Asthma	493.01	Asthma with allergic rhinitis and status asthmaticus	1
Asthma	493.01	Asthma with status asthmaticus in pediatric patient	1
Asthma	493.01	Asthma, extrinsic, with status asthmaticus	1
Asthma	493.02	Extrinsic asthma with exacerbation	1831
Asthma	493.02	Extrinsic asthma, with acute exacerbation	10
Asthma	493.02	Asthma exacerbation, allergic	5
Asthma	493.02	Asthma with acute exacerbation in pediatric patient	3
Asthma	493.02	Asthma exacerbation, allergic, moderate persistent	2
Asthma	493.02	Asthma, extrinsic with exacerbation	2
Asthma	493.02	Moderate persistent asthma with acute exacerbation in pediatric patient	2
Asthma	493.02	Asthma exacerbation, allergic, severe persistent	1
Asthma	493.1	Intrinsic asthma	261
Asthma	493.10	Intrinsic asthma, unspecified	182
Asthma	493.10	Asthma, cold induced	19
Asthma	493.10	Cold-induced asthma	11
Asthma	493.10	Asthma, intrinsic, without status asthmaticus	2
Asthma	493.10	Asthma occurring only with upper respiratory infection	1
Asthma	493.10	Asthma, endogenous	1
Asthma	493.10	Asthma, intrinsic	1
Asthma	493.10	Cold-induced asthma without complication	1
Asthma	493.10	Intrinsic asthma	1
Asthma	493.11	Intrinsic asthma with status asthmaticus	2
Asthma	493.11	Status asthmaticus, intrinsic	1
Asthma	493.12	Intrinsic asthma with exacerbation	6

Table S1 ICD9 codes by diagnosis group (continued)

Diagnosis Group	ICD9 Code	Diagnosis Name	n
Asthma	493.12	Asthma exacerbation, non-allergic	1
Asthma	493.12	Intrinsic asthma with acute exacerbation	1
Asthma	493.12	Mild intermittent intrinsic asthma with exacerbation	1
Asthma	493.20	Chronic obstructive asthma, unspecified	3
Asthma	493.20	Asthma with chronic obstructive pulmonary disease (COPD)	1
Asthma	493.20	Asthma, chronic obstructive, without status asthmaticus	1
Asthma	493.22	Chronic obstructive asthma with exacerbation	4
Asthma	493.81	Exercise induced bronchospasm	1553
Asthma	493.81	Exercise-induced asthma	1276
Asthma	493.81	Asthma, exercise induced	228
Asthma	493.81	Exercise-induced bronchospasm	118
Asthma	493.81	Bronchospasm, exercise-induced	16
Asthma	493.81	Exercise-induced bronchoconstriction	6
Asthma	493.81	Exertional asthma	6
Asthma	493.81	Mild exercise-induced asthma	4
Asthma	493.81	Exercise-induced asthma with acute exacerbation	2
Asthma	493.82	Cough variant asthma	416
Asthma	493.82	Asthma, cough variant	43
Asthma	493.9	Mild intermittent asthma	64381
Asthma	493.90	Mild persistent asthma	27104
Asthma	493.90	Moderate persistent asthma	8073
Asthma	493.90	Asthma	7420
Asthma	493.90	Unspecified asthma(493.90)	3551
Asthma	493.90	Severe persistent asthma	841
Asthma	493.90	Asthma, mild intermittent	435
Asthma	493.9	Unspecified asthma	244
Asthma	493.90	Asthma, chronic	191
Asthma	493.90	Asthma without status asthmaticus	184
Asthma	493.90	Asthma, mild persistent	169
Asthma	493.90	Intermittent asthma	90
Asthma	493.90	Mild asthma	75
Asthma	493.90	Asthma, moderate persistent	72
Asthma	493.90	Asthma, intermittent	51
Asthma	493.90	Moderate intermittent asthma	39
Asthma	493.90	Asthma, mild intermittent, well-controlled	23
Asthma	493.90	Allergy-induced asthma	19
Asthma	493.90	Asthma, severe persistent	16
Asthma	493.90	Mild intermittent asthma without complication	15
Asthma	493.90	Mild persistent asthma without complication	13
Asthma	493.90	Asthma, moderate persistent, well-controlled	12
Asthma	493.90	Allergic asthma	11
Asthma	493.90	Asthma, mild	11
Asthma	493.90	Asthma, moderate persistent, poorly-controlled	10
Asthma	493.90	Asthma in remission	9
Asthma	493.90	Asthma, allergic	9
Asthma	493.90	Persistent asthma	9
Asthma	493.90	Moderate asthma	8
Asthma	493.90	Asthma, currently inactive	7
Asthma	493.90	Seasonal asthma	6
Asthma	493.90	Asthma, persistent	5
Asthma	493.90	Poorly controlled persistent asthma	4
Asthma	493.90	Asthma night-time symptoms	3
Asthma	493.90	Asthma, currently active	3
Asthma	493.90	Asthma, intermittent controlled	3
Asthma	493.90	Asthma, moderate	3
Asthma	493.90	Asthmatic bronchitis	3
Asthma	493.90	Bronchial asthma	3

Table S1 ICD9 codes by diagnosis group (continued)

Diagnosis Group	ICD9 Code	Diagnosis Name	n
Asthma	493.90	Mild persistent allergic asthma without complication	3
Asthma	493.90	Moderate persistent asthma without complication	3
Asthma	493.90	Asthma, currently dormant	2
Asthma	493.90	Asthma, mild intermittent, poorly controlled	2
Asthma	493.90	Asthma, severe persistent, well-controlled	2
Asthma	493.90	Chronic asthma	2
Asthma	493.90	Intermittent asthma, well controlled	2
Asthma	493.90	Mild intermittent asthma, uncomplicated	2
Asthma	493.90	Moderate persistent allergic asthma without complication	2
Asthma	493.90	Severe asthma	2
Asthma	493.90	Severe persistent asthma, well-controlled	2
Asthma	493.90	Acute asthma	1
Asthma	493.90	Allergic bronchitis	1
Asthma	493.90	Asthma due to environmental allergies	1
Asthma	493.90	Asthma with bronchitis	1
Asthma	493.90	Asthma, acute	1
Asthma	493.90	Asthma, intermittent not controlled	1
Asthma	493.90	Asthma, persistent not controlled	1
Asthma	493.90	Asthma, severe	1
Asthma	493.90	Asthma, well controlled	1
Asthma	493.90	Brittle asthma	1
Asthma	493.90	Bronchitis, asthmatic	1
Asthma	493.90	Inactive asthma	1
Asthma	493.90	Mild intermittent asthma in adult without complication	1
Asthma	493.90	Mild intermittent asthma without status asthmaticus without complication	1
Asthma	493.90	Mild persistent asthma in adult without complication	1
Asthma	493.90	Mild persistent asthma, well controlled	1
Asthma	493.90	Nocturnal asthma	1
Asthma	493.90	Rapid onset asthma	1
Asthma	493.90	Uncontrolled moderate persistent asthma	1
Asthma	493.91	Status asthmaticus	2073
Asthma	493.91	Asthma with status asthmaticus	1017
Asthma	493.91	Unspecified asthma, with status asthmaticus	26
Asthma	493.91	Mild intermittent asthma with status asthmaticus	20
Asthma	493.91	Mild persistent asthma with status asthmaticus	20
Asthma	493.91	Moderate persistent asthma with status asthmaticus	15
Asthma	493.91	Acute severe asthma	5
Asthma	493.91	Asthma, with status asthmaticus	5
Asthma	493.91	Asthma with severe asthma attack	4
Asthma	493.91	Severe persistent asthma with status asthmaticus	4
Asthma	493.91	Status asthmaticus, allergic	2
Asthma	493.92	Unspecified asthma, with exacerbation	7275
Asthma	493.92	Asthma exacerbation	1967
Asthma	493.92	Asthma with acute exacerbation	1465
Asthma	493.92	Asthma flare	287
Asthma	493.92	Mild intermittent asthma with acute exacerbation	193
Asthma	493.92	Mild persistent asthma with acute exacerbation	129
Asthma	493.92	Asthma with exacerbation	80
Asthma	493.92	Acute asthma exacerbation	40
Asthma	493.92	Asthma attack	34
Asthma	493.92	Acute asthma flare	21
Asthma	493.92	Moderate persistent asthma with exacerbation	18
Asthma	493.92	Asthma exacerbation, mild	9
Asthma	493.92	Moderate persistent asthma with acute exacerbation	8
Asthma	493.92	Severe persistent asthma with acute exacerbation	5
Asthma	493.92	Asthma exacerbation attacks	4
Asthma	493.92	Exacerbation of asthma	2

Table S1 ICD9 codes by diagnosis group (continued)

Diagnosis Group	ICD9 Code	Diagnosis Name	n
Asthma	493.92	Mild intermittent asthma with exacerbation	2
Asthma	493.92	Mild persistent asthma with exacerbation	2
Asthma	493.92	Severe persistent asthma with exacerbation	2
Asthma	493.92	Acute severe exacerbation of asthma	1
Asthma	493.92	Asthma, intermittent with acute exacerbation	1
Asthma	493.92	Mild persistent allergic asthma with acute exacerbation	1
Rhinitis	477.0	Allergic rhinitis due to pollen	3146
Rhinitis	477	Allergic rhinitis	592
Rhinitis	477.0	Pollen allergies	189
Rhinitis	477.0	Pollen allergy	170
Rhinitis	477.0	Rhinitis due to pollen	81
Rhinitis	477.0	Allergy to pollen	79
Rhinitis	477.0	Allergy to trees	69
Rhinitis	477.1	Allergic rhinitis due to food	16
Rhinitis	477.2	Allergic rhinitis due to animal hair and dander	557
Rhinitis	477.2	Allergic rhinitis due to animal (cat) (dog) hair and dander	385
Rhinitis	477.2	Allergy to dogs	185
Rhinitis	477.2	Allergic to dogs	129
Rhinitis	477.2	Animal dander allergy	96
Rhinitis	477.2	Allergy to dog dander	90
Rhinitis	477.2	Dog allergy due to both airborne and skin contact	61
Rhinitis	477.2	Allergic rhinitis due to animal hair or dander	50
Rhinitis	477.2	Dander (animal) allergy	36
Rhinitis	477.2	Allergy to animal dander	31
Rhinitis	477.2	Allergy to animals	30
Rhinitis	477.2	Allergic rhinitis due to animals	22
Rhinitis	477.2	Allergic to animal dander	15
Rhinitis	477.2	Cat allergy due to both airborne and skin contact	9
Rhinitis	477.2	Allergic rhinitis due to cat hair	7
Rhinitis	477.2	Allergic rhinitis due to animal dander	5
Rhinitis	477.2	Allergic rhinitis due to cats	5
Rhinitis	477.2	Allergic rhinitis due to dogs	4
Rhinitis	477.2	Rhinitis, allergic, due to animal hair or dander	2
Rhinitis	477.8	Allergic rhinitis due to other allergen	2066
Rhinitis	477.8	Cat allergies	310
Rhinitis	477.8	Allergy to cats	290
Rhinitis	477.8	Allergic to cats	144
Rhinitis	477.8	Dust allergy	76
Rhinitis	477.8	Allergy to dust	32
Rhinitis	477.8	Environmental and seasonal allergies	27
Rhinitis	477.8	Allergic to pets	17
Rhinitis	477.8	Pet allergy	17
Rhinitis	477.8	Cat allergy, airborne	13
Rhinitis	477.8	Allergic rhinitis due to dust	10
Rhinitis	477.8	Allergy to feathers	10
Rhinitis	477.8	Allergic reaction to inhaled dust	5
Rhinitis	477.8	Allergic rhinitis due to house dust mite	5
Rhinitis	477.8	Airborne cat allergy	4
Rhinitis	477.8	Allergic rhinitis NEC	1
Rhinitis	477.8	Rhinitis, allergic to other allergen	1
Rhinitis	477.8	Smoke hypersensitivity	1
Rhinitis	477.9	Allergic rhinitis, cause unspecified	46860
Rhinitis	477.9	Allergic rhinitis	28677
Rhinitis	477.9	Seasonal allergies	8310
Rhinitis	477.9	Allergic rhinitis due to allergen	839
Rhinitis	477.9	Seasonal allergic rhinitis	693
Rhinitis	477.9	Allergic rhinitis, seasonal	368

Table S1 ICD9 codes by diagnosis group (continued)

Diagnosis Group	ICD9 Code	Diagnosis Name	n
Rhinitis	477.9	Allergic rhinoconjunctivitis	342
Rhinitis	477.9	Seasonal allergic reaction	107
Rhinitis	477.9	Seasonal rhinitis	80
Rhinitis	477.9	Allergic conjunctivitis and rhinitis	76
Rhinitis	477.9	AR (allergic rhinitis)	70
Rhinitis	477.9	Perennial allergic rhinitis	56
Rhinitis	477.9	Hayfever	39
Rhinitis	477.9	Rhinitis, allergic	33
Rhinitis	477.9	Hay fever	26
Rhinitis	477.9	Atopic rhinitis	23
Rhinitis	477.9	Allergic rhinoconjunctivitis, seasonal and perennial	18
Rhinitis	477.9	Allergic shiners	18
Rhinitis	477.9	Allergic rhinoconjunctivitis of both eyes	15
Rhinitis	477.9	Vasomotor rhinitis	14
Rhinitis	477.9	Seasonal and perennial allergic rhinoconjunctivitis	12
Rhinitis	477.9	Allergic rhinosinusitis	7
Rhinitis	477.9	Multiple respiratory allergies	5
Rhinitis	477.9	Allergic sinusitis	4
Rhinitis	477.9	Nasal inflammation due to allergen	3
Rhinitis	477.9	Seasonal and perennial allergic rhinitis	3
Rhinitis	477.9	Allergic conjunctivitis of both eyes and rhinitis	2
Rhinitis	477.9	Allergic rhinitis, mild	2
Rhinitis	477.9	Allergic rhinitis with postnasal drip	1
Rhinitis	477.9	Chronic allergic rhinitis	1
Rhinitis	477.9	Mild allergic rhinitis	1
Rhinitis	477.9	Nonallergic vasomotor rhinitis	1
Rhinitis	477.9	Non-allergic vasomotor rhinitis	1
Rhinitis	477.9	Non-seasonal allergic rhinitis	1
Rhinitis	477.9	Perennial allergic rhinitis with seasonal variation	1
