# Supplementary material for: The epidemiologic characteristics of healthcare provider-diagnosed eczema, asthma, allergic rhinitis, and food allergy in children: a retrospective cohort study
Source: BMC Pediatr. 2016 Aug 20;16:133. doi: 10.1186/s12887-016-0673-z (PMC4992234; doi:10.1186/s12887-016-0673-z)
Supplement: Additional file 2: Table S2. — Food allergy codes by allergen; A table of food allergy codes used to define allergy to each food allergen. (PDF 258 kb) [file 12887_2016_673_MOESM2_ESM.pdf]

Table S2 Food allergy codes by allergen

Diagnosis Group	Allergen	Allergy code	n
Food Allergy	Almond	Almond	903
Food Allergy	Almond	Almond oil, bitter	195
Food Allergy	Almond	Almond meal	30
Food Allergy	Brazil nut	Brazil nut	170
Food Allergy	Cashew	Cashew	1,646
Food Allergy	Cashew	OTHER: ADD COMMENT:Cashew Nuts	1
Food Allergy	Egg	Egg	15,765
Food Allergy	Egg	Eggs	124
Food Allergy	Egg	Egg yolk	98
Food Allergy	Egg	Eggshell membrane (chicken)	84
Food Allergy	Egg	Egg lecithin	23
Food Allergy	Egg	E-egg white-soybean lecithin	4
Food Allergy	Egg	Egg phospholipids	4
Food Allergy	Egg	Egg-pro	2
Food Allergy	Egg	OTHER: ADD COMMENT:Dairy, Eggs	1
Food Allergy	Egg	OTHER: ADD COMMENT:Egg/milk	1
Food Allergy	Egg	OTHER: ADD COMMENT:Eggs, Chocolate	1
Food Allergy	Egg	OTHER: ADD COMMENT:Eggs/milk	1
Food Allergy	Egg	OTHER: ADD COMMENT:Peanut, Eggs, Soy	1
Food Allergy	Egg	OTHER: ADD COMMENT:Peanuts, Eggs, Fish, Milk	1
Food Allergy	Egg	OTHER: ADD COMMENT:Peanuts,egg,dairy	1
Food Allergy	Fish	Fish	2932
Food Allergy	Fish	Fish products	520
Food Allergy	Fish	Fish, with scales	408
Food Allergy	Fish	Salmon	287
Food Allergy	Fish	Tuna	226
Food Allergy	Fish	Fish allergy	190
Food Allergy	Fish	Fish oil	29
Food Allergy	Fish	Whitefish	24
Food Allergy	Fish	Fish-derived products	13
Food Allergy	Fish	OTHER: ADD COMMENT:Fish	2
Food Allergy	Fish	DHA-E-EPA-fish oil-omega 3 fatty acids	1
Food Allergy	Fish	OTHER: ADD COMMENT:Peanuts, Eggs, Fish, Milk	1
Food Allergy	Fish	OTHER: ADD COMMENT:Salmon,pastachios Water Mellon	1
Food Allergy	Fish	OTHER: ADD COMMENT:Tuna	1
Food Allergy	Fish	RA fish	1
Food Allergy	Fish	Salmon oil-black currant-Vit E	1
Food Allergy	Hazelnut	Hazelnut (filbert)	718
Food Allergy	Milk	Milk (cow's)	20636
Food Allergy	Milk	Milk based formula	71
Food Allergy	Milk	Milk-related compounds	9
Food Allergy	Milk	OTHER: ADD COMMENT:Milk	5
Food Allergy	Milk	Cow's milk	4
Food Allergy	Milk	OTHER: ADD COMMENT:Cow's Milk	2
Food Allergy	Milk	OTHER: ADD COMMENT:Egg/milk	1
Food Allergy	Milk	OTHER: ADD COMMENT:Eggs/milk	1
Food Allergy	Milk	OTHER: ADD COMMENT:Milk /products	1
Food Allergy	Milk	OTHER: ADD COMMENT:Milk Intolerant	1
Food Allergy	Milk	OTHER: ADD COMMENT:Milk Products	1
Food Allergy	Milk	OTHER: ADD COMMENT:Milk Sensitivity	1
Food Allergy	Milk	OTHER: ADD COMMENT:Peanuts, Eggs, Fish, Milk	1
Food Allergy	Milk	OTHER: ADD COMMENT:Soy Milk	1
Food Allergy	Milk	OTHER: ADD COMMENT:Soy Milk,	1
Food Allergy	Peanut	Peanut	22,269
Food Allergy	Peanut	Peanut oil	1,214
Food Allergy	Peanut	Peanuts	313
Food Allergy	Peanut	Peanut butter	190

Table S2 Food allergy codes by allergen (continued)

Diagnosis Group	Allergen	Allergy code	n
Food Allergy	Peanut	OTHER: ADD COMMENT:Peanut Butter	12
Food Allergy	Peanut	OTHER: ADD COMMENT:Peanutbutter	2
Food Allergy	Peanut	OTHER: ADD COMMENT:Peanut Butter Allergy	1
Food Allergy	Peanut	OTHER: ADD COMMENT:Peanut, Eggs, Soy	1
Food Allergy	Peanut	OTHER: ADD COMMENT:Peanuts	1
Food Allergy	Peanut	OTHER: ADD COMMENT:Peanuts, Eggs, Fish, Milk	1
Food Allergy	Peanut	OTHER: ADD COMMENT:Peanuts, Nuts	1
Food Allergy	Peanut	OTHER: ADD COMMENT:Peanuts,dairy,pollen,ragweed	1
Food Allergy	Peanut	OTHER: ADD COMMENT:Peanuts,egg,dairy	1
Food Allergy	Pecan	Pecan	714
Food Allergy	Pecan	OTHER: ADD COMMENT:Pecans,apricots	1
Food Allergy	Pistachio	Pistachio	970
Food Allergy	Pistachio	OTHER: ADD COMMENT:Pistachios	1
Food Allergy	Sesame	Sesame seed	2,885
Food Allergy	Sesame	Sesame oil	73
Food Allergy	Shellfish	Shellfish allergy	12760
Food Allergy	Shellfish	Crab	257
Food Allergy	Shellfish	Shellfish	188
Food Allergy	Shellfish	Clam	123
Food Allergy	Shellfish	Shrimp	67
Food Allergy	Shellfish	Lobster	27
Food Allergy	Shellfish	OTHER: ADD COMMENT:Shellfish	21
Food Allergy	Shellfish	Seafood: shellfish	18
Food Allergy	Shellfish	OTHER: ADD COMMENT:Shrimp	13
Food Allergy	Shellfish	Scallop	10
Food Allergy	Shellfish	OTHER: ADD COMMENT:Crabs	4
Food Allergy	Shellfish	Shellfish-Derived Products	3
Food Allergy	Shellfish	OTHER: ADD COMMENT:Scallop	1
Food Allergy	Shellfish	OTHER: ADD COMMENT:To Shrimp-- Hives On Face	1
Food Allergy	Soy	Soy	6919
Food Allergy	Soy	Soy formula	229
Food Allergy	Soy	Soybean allergy	123
Food Allergy	Soy	Soybeans	63
Food Allergy	Soy	Soybean oil	47
Food Allergy	Soy	Soybean (soy) Protein	17
Food Allergy	Soy	Soy Allergy	15
Food Allergy	Soy	Soy fiber	9
Food Allergy	Soy	Soy flour	3
Food Allergy	Soy	FD&C yellow #6 AL lake-guar gum-soy oil	1
Food Allergy	Soy	KDC:borage oil+linseed oil+oleic acid+soybean oil+vitamin E	1
Food Allergy	Soy	OTHER: ADD COMMENT:Soy Products	1
Food Allergy	Soy	OTHER: ADD COMMENT:Soy Milk	1
Food Allergy	Soy	OTHER: ADD COMMENT:Soy Milk,	1
Food Allergy	Soy	Silybum-soy-soy lecithin	1
Food Allergy	Soy	Soy germ	1
Food Allergy	Treenut	OTHER: ADD COMMENT:Tree Nuts	1
Food Allergy	Treenut	OTHER: ADD COMMENT:Treenuts	1
Food Allergy	Walnut	Walnut	1,784
Food Allergy	Walnut	OTHER: ADD COMMENT:Walnut	1
Food Allergy	Wheat	Wheat	3,902
Food Allergy	Wheat	Wheat bran	10
Food Allergy	Wheat	OTHER: ADD COMMENT:Dairy And Wheat Products	1
