# Supplementary material for: The epidemiologic characteristics of healthcare provider-diagnosed eczema, asthma, allergic rhinitis, and food allergy in children: a retrospective cohort study
Source: BMC Pediatr. 2016 Aug 20;16:133. doi: 10.1186/s12887-016-0673-z (PMC4992234; doi:10.1186/s12887-016-0673-z)
Supplement: Additional file 3: Table S3. — ICD9 codes excluded from our analysis; A table of ICD9 codes that were excluded from our analysis. (PDF 210 kb) [file 12887_2016_673_MOESM3_ESM.pdf]

Table S3 ICD9 codes excluded from our analysis

ICD9 Code	Diagnosis Name
493.10	Post viral asthma
493.10	Post viral RAD (reactive airway disease)
493.10	Post-viral reactive airway disease
493.81	Exercise-induced RAD (reactive airway disease)
493.81	Exercise-induced reactive airway disease
493.90	RAD (reactive airway disease)
493.90	RAD (reactive airway disease) with wheezing
493.90	Reactive airway disease
493.90	Reactive airway disease with wheezing
493.90	Reactive airways dysfunction syndrome
493.92	Exacerbation of RAD (reactive airway disease)
493.92	Exacerbation of reactive airway disease
