# Supplementary material for: The epidemiologic characteristics of healthcare provider-diagnosed eczema, asthma, allergic rhinitis, and food allergy in children: a retrospective cohort study
Source: BMC Pediatr. 2016 Aug 20;16:133. doi: 10.1186/s12887-016-0673-z (PMC4992234; doi:10.1186/s12887-016-0673-z)
Supplement: Additional file 4: Table S4. — Percent confirmed diagnosis on chart review; A table indicating the percent confirmed diagnosis on manual chart review. (PDF 199 kb) [file 12887_2016_673_MOESM4_ESM.pdf]

Table S4 Percent confirmed diagnosis on chart review

Condition	Frequency, %
Eczema	90
Food allergy	88
Asthma	90
Rhinitis	100
All diagnoses	92
