# Supplementary material for: The epidemiologic characteristics of healthcare provider-diagnosed eczema, asthma, allergic rhinitis, and food allergy in children: a retrospective cohort study
Source: BMC Pediatr. 2016 Aug 20;16:133. doi: 10.1186/s12887-016-0673-z (PMC4992234; doi:10.1186/s12887-016-0673-z)
Supplement: Additional file 5: Table S5. — Analysis of asthma medication prescriptions; A table summarizing our analysis of asthma medication prescription practices and diagnosis codes. (PDF 208 kb) [file 12887_2016_673_MOESM5_ESM.pdf]

Table S5 Analysis of asthma medication prescriptions

Group	Frequency, % (n)	
	Birth	Cross-sectional
Whole cohort	(29,662)	(333,200)
Patients with 493 ICD-9 code	22.4 (6,659)	21.8 (72,534)
Patients with asthma prescriptions	30.3 (8,984)	24.5 (81,667)
Patients with 493 ICD-9 code & prescriptions	21.6 (6,395)	18.4 (61,328)
